# Splenic architecture disruption and parasite-induced splenocyte activation and anergy in *Plasmodium falciparum*-infected *Saimiri sciureus* monkeys

**DOI:** 10.1186/s12936-015-0641-3

**Published:** 2015-03-25

**Authors:** Francisco A Alves, Marcelo Pelajo-Machado, Paulo RR Totino, Mariana T Souza, Evonnildo C Gonçalves, Maria Paula C Schneider, José APC Muniz, Marco A Krieger, Marcia CR Andrade, Cláudio Tadeu Daniel-Ribeiro, Leonardo JM Carvalho

**Affiliations:** Laboratório de Pesquisa em Malária, Instituto Oswaldo Cruz (IOC), Fiocruz, Rio de Janeiro, Brazil; Laboratório de Patologia, Instituto Oswaldo Cruz (IOC), Fiocruz, Rio de Janeiro, Brazil; Laboratório de Imunobiologia, Universidade Federal do Pará (UFPA), Belém, Brazil; Laboratório de Tecnologia Biomolecular, Universidade Federal do Pará (UFPA), Belém, Brazil; Laboratório de Polimorfismo de DNA, Universidade Federal do Pará (UFPA), Belém, Brazil; Centro Nacional de Primatas (Cenp), SVS, Belém, Brazil; Instituto Carlos Chagas de Biologia Molecular, Curitiba, Brazil; Serviço de Criação de Primatas Não-Humanos, CECAL-Fiocruz, Rio de Janeiro, Brazil

**Keywords:** Malaria, *Saimiri*, *Aotus*, *Plasmodium falciparum*, Cytokines, Spleen

## Abstract

**Background:**

The understanding of the mechanisms of immunity in malaria is crucial for the rational development of interventions such as vaccines. During blood stage infection, the spleen is considered to play critical roles in both immunity and immunopathology of *Plasmodium falciparum* infections.

**Methods:**

*Saimiri sciureus* monkeys were inoculated with blood stages of *P. falciparum* (FUP strain) and spleens removed during acute disease (days 7 and 13 of infection) and during convalescence (15 days after start of chloroquine treatment). Cytokine (IFN**γ**, TNFα, IL2, IL6, IL10, and IL12) responses of splenocytes stimulated with *P. falciparum*-parasitized red blood cells were assessed by real-time PCR using specific *Saimiri* primers, and histological changes were evaluated using haematoxylin-eosin and Giemsa-stained slides.

**Results:**

Early during infection (day 7, 1-2% parasitaemia), spleens showed disruption of germinal centre architecture with heavy B-cell activation (centroblasts), and splenocytes showed increased expression of IFNγ, IL6 and IL12 upon *in vitro* stimuli by *P. falciparum*-parasitized red blood cells (pRBC). Conversely, 15 days after treatment of blood stage infection with chloroquine, splenocytes showed spontaneous *in vitro* expression of TNFα, IL2, IL6, IL10, and IL12, but not IFNγ, and stimulation with *P. falciparum* pRBC blocked the expression of all these cytokines. During the acute phase of infection, splenic disarray with disorganized germinal centres was observed. During convalescence, spleens of the chloroquine-treated animals showed white pulp hyperplasia with extensive lymphocyte activation and persistency of heavily haemozoin-laden macrophages throughout the red pulp.

**Conclusions:**

Inability to eliminate haemozoin is likely involved in the persistent lymphocyte activation and in the anergic responses of *Saimiri* splenocytes to *P. falciparum* pRBC, with important negative impact in immune responses and implications for the design of malaria vaccine.

## Background

Malaria infections induce complex and not still completely understood immune responses. Individuals continuously exposed to infection in areas of intense malaria transmission take many years to develop effective clinical and parasitological immunity, although adults may develop effective responses faster than children [1-4]. It appears, therefore, that immune responses to sporozoite, liver and blood stages of *Plasmodium* as well to heterologous antigens, such as vaccines against immuno-preventable diseases, are defective [5]. Despite being of limited efficacy against the parasite or its toxic products, immune responses to malaria infections can be deleterious, causing immunopathology, which is believed to play a role in complications such as cerebral malaria [6,7]. The limited knowledge of the mechanisms of immunity and pathogenesis in malaria creates difficulties for the development of rational preventative and therapeutic interventions, such as vaccines [8].

The neotropical monkeys *Aotus* and *Saimiri* are the non-human primates recommended by the World Health Organization for experimental malaria research [9] and represent the closest animal models to the human infection owing to their unique ability to develop a reproducible parasitaemia when inoculated with blood stages of *Plasmodium falciparum* or *Plasmodium vivax*. These features make them particularly useful in pre-clinical trials of potential malaria vaccines, as well in studies of pathogenesis of the disease [10-13]. However, studies with these animals are often limited by the lack of specific reagents and molecular tools allowing, for instance, reliable evaluation of immune responses. The partial nucleotide sequence of 13 cytokine genes of *Saimiri sciureus* and *Aotus infulatus* had been previously described [14]. In the present study primers specific for the *Saimiri* cytokines IFNγ, TNFα, IL2, IL6, IL10, and IL12 were designed and used to study splenic cellular responses during and after blood stage *P. falciparum* infection in *S. sciureus* monkeys.

## Methods

### Animals

*Saimiri sciureus* monkeys were bred and housed at the National Primate Centre, Secretaria de Vigilância em Saúde (SVS, Health Surveillance Secretary) in Belém, and at the Department of Primatology of the Centro de Criação de Animais de Laboratório (CECAL, Centre for Breeding of Laboratory Animals), Fiocruz, Rio de Janeiro, Brazil. All animals used in this study were adults born in captivity. The experimental protocols were reviewed and approved by the local Ethical Committees for Animal Use.

### Design and validation of specific primers

The primers for cytokine amplification by quantitative real time PCR (qRT-PCR) were designed using as template the previously described conserved genomic sequences between *S. sciureus* and *A. infulatus* [14]. The primers used were as follows: IFNγ: 5′-TTTCTTAAACATTTTGAGGACTTGGA-3′ (sense) and 5′-AAGGAGATAATCTGGCTCTGCATT-3′ (antisense); TNFα: 5′-CTCTTCTGCCTGCTGCACTTC-3′ (sense) and 5′-AAGTCCCTGGAGGACTGCTCTT-3′ (antisense); IL2: 5′-GTGCACCTACTTCAAGTTCTACAAAGA-3′ (sense) and 5′-CATCTGTAAGTCCAGCAGTAAATGC-3′ (antisense); IL6: 5′-TGGCAGAAAAAGATGGATGCT-3′ (sense) and 5′-CTCCAAAAGACCAGTGGTGATTT-3′ (antisense); IL10: 5′-GCAGTGGCGCAGGTGAA-3′ (sense) and 5′-GGCTTTGTAGACGCCTTTCTCTT-3′ (antisense); IL12 (IL12p40): 5′-AGGTCTTAGGCTCTGGCAAAAA-3′ (sense) and 5′-TGGCCAGCATCTCCAACTCT-3′ (antisense); and β-actin (internal control of reaction): 5′-CACCACACCTTCTACAATGAG-3′ (sense) and 5′-GTCTCAAACATGATCTGGGTC-3′ (antisense). The primers above were designed based on the following *Saimiri* and *Aotus* GenBank sequences: IFNγ: DQ989367 (*Saimiri*) and DQ989366 (*Aotus*); TNFα: DQ989365 (*Saimiri*) and DQ989364 (*Aotus*); IL2: DQ989369 (*Saimiri*) and DQ989368 (*Aotus*); IL6: DQ985387 (*Saimiri*) and DQ985386 (*Aotus*); IL10: DQ989357 (*Saimiri*); IL12p40: DQ989358 (*Saimiri*) and DQ989359 (*Aotus*).

To validate the primers, blood (4 mL) was withdrawn from the femoral vein of healthy adult *S. sciureus* monkeys into EDTA-containing Vacutainer tubes. Peripheral blood mononuclear cells (PBMC) were separated by density gradient centrifugation using Ficoll-Hypaque 1077 (Sigma, St Louis, USA) and washed three times with RPMI (Sigma). PBMC (2×10^6^ cells/well) were cultured in RPMI 1640 medium supplemented with 10% foetal calf serum with or without 500 ng/mL ionomycin and 50 ng/mL phorbol 12-myristate 13-acetate (PMA) (all from Sigma). The plates were incubated for two, four, six, eight, 12, and 18 hours at 37°C in 5% CO_2_. At each time point, cells were harvested and lysed for RNA extraction (see below).

### Purification of total RNA and cDNA synthesis

Total RNA was isolated from cells using *RNeasy kit* (Qiagen) to a final volume of 50 mL according to the manufacturer’s protocol. Then, the RNA was concentrated using YM-30 ultracel Microcon (Millipore), resuspended to 10 mL and transcribed to cDNA using High-Capacity kit (Applied Biosystems). Briefly, the reaction contained 10 mL total RNA (initial amount of 1 mg), 1X RT Buffer, 1X dNTP mix (100 mM), 1X RT Random Primers, 2.5U MultiScribe^TM^ Reverse Transcriptase and 3.2 ml nuclease-free water in a total volume of 20 mL. The reaction mixture was incubated at 25°C for 10 min, followed by heating at 37°C for 120 min and at 85°C for 5 sec, concentrated with YM-30 ultracel Microcon (Millipore), and resuspended for final volume of 500 mL, aliquoted (2 ng/mL) and maintained at −20°C.

### Quantitative real-time PCR and reagent mix preparation

The qRT-PCR was carried out in a 20-mL final volume containing: i) 4 mL H_2_O; ii) 1 mL forward and reverse primer mix (4 pmol/mL); iii) 5 mL cDNA; and, iv) 10 mL SYBR Green Master Mix (Applied Biosystems). The reaction contained three stages: i) 50°C for 2 min to activate the Uracil-DNA N-glycosylase (UNG) enzyme; ii) 95°C for 10 min to activate the TaqGold enzyme; and, iii) 45 cycles at 95°C for 15 sec, at 60°C for 20 sec and at 72°C for 1 min. Results were expressed in fold-change in relation to the calibrator sample (extracted RNA without mitogen stimulation).

### *Plasmodium falciparum* infection and follow up

Nine intact (non-splenectomized) *S. sciureus* monkeys were used in the infection experiments (six infected and three controls). Six monkeys were injected intravenously with 5x10^7^*P. falciparum* (FUP)-pRBC with a predominance of ring and young trophozoite stages obtained from an infected splenectomized *S. sciureus*. Parasitaemia was followed up daily by examination of Giemsa-stained thin smears of blood obtained from the footpad. Two infected *Saimiri* monkeys were treated with chloroquine (three daily doses of 10 mg/kg) on day (d) 7 (parasitaemia of 1-2%) and four *Saimiri* monkeys were treated on d13 of infection (parasitaemia over 10%). Monkeys were subjected to splenectomy right before chloroquine treatment (two monkeys on d7 and two monkeys on d13) or 15 days after the start of chloroquine treatment (two monkeys on d28).

### *Saimiri* splenocytes

*Plasmodium falciparum*-infected *Saimiri* monkeys were subjected to splenectomy at the time points described above (d7, d13 and d28). One uninfected control *Saimiri* monkey was also splenectomized at each day (d7, d13 and d28, total of three control animals). Splenectomy was performed under anaesthesia with ketamine (150 mg/kg) plus xylazine (10 mg/kg), in an aseptic surgical room by a trained veterinarian. After spleen removal and vascular resections, the wound was sutured and the animals allowed to recover under close supervision of trained staff. After recovery from surgery, monkeys were treated with chloroquine. Right after removal, a piece of the spleen was immersed and kept in formalin for histological analysis and a second piece was conditioned in sterile RPMI 1640 medium supplemented with 10% foetal calf serum and handled in a laminar flow cabinet. Spleens were gently fragmented between glass microscope slides and the splenocyte suspension was layered on Ficoll-hypaque 1077 (Sigma) gradient. Splenocytes were cultured *in vitro* under antigenic stimulation with *P. falciparum-*parasitized red blood cells (pRBC) (see below).

### *In vitro Plasmodium falciparum* culture

*Plasmodium falciparum* (FCR3 strain) was maintained *in vitro* in continuous culture according to the method described by Trager and Jensen [15] under an atmosphere of 5% CO_2_, 5% O_2_ and 90% N_2_ (White Martins) using O+ human RBC in RPMI-1640 medium (Sigma) supplemented with 25 mM Hepes (Sigma), 0.2% glucose (Sigma), 23 mM sodium bicarbonate (Sigma) and 40 mg/L gentamycin (Gibco).

### *In vitro* splenocyte stimulation

Freshly isolated splenocytes (2×10^6^ cells/well) were layered in 24-well tissue culture plates (Corning, NY, USA) in HEPES-buffered 1640 RPMI supplemented with 10% heat-inactivated foetal calf serum (Sigma) and co-cultured with asynchronous *P. falciparum*-pRBC or normal, uninfected RBC, at a proportion of 20 pRBC (or RBC) per splenocyte. Cells were incubated at 37°C for six hours in an atmosphere of 5% CO_2_. After culture, cells were harvested and lysed for RNA extraction, cDNA synthesis and qRT-PCR as described above. Results were expressed in fold-change in relation to non-stimulated splenocytes from control uninfected monkeys.

### Histological analysis

Spleen fragments were fixed in Carson’s modified Millonig’s phosphate-buffered formalin, pH 7.4 [16]. The specimens were processed with increasing ethanol concentrations (70, 95 and 100%, for one hour each), cleared in xylene (two hours), and paraffin embedded. Sections of 5 μm thickness were dewaxed with xylene (three times), hydrated with ethanol (three times each in 100, 95, 70, and 50%) and stained with Lennert’s Giemsa [17,18]. Slides were analysed by bright-field microscopy (Zeiss Axiovert 200 M) and images captured with a digital camera (Zeiss Axiocam HRc) and processed with the ZoomBrowser EX software (Canon).

### Statistical analysis

All statistical analyses were performed using a statistical software package (Prism 5.0, Graphpad). A two-way analysis of variance (ANOVA) test with Bonferroni post-hoc analysis was used to determine the significance of differences in cytokine expression on d7 and 28. A p value <0.05 was considered significant.

## Results

### Validation of *Saimiri* cytokine primers for qRT-PCR

**Figure 1:**
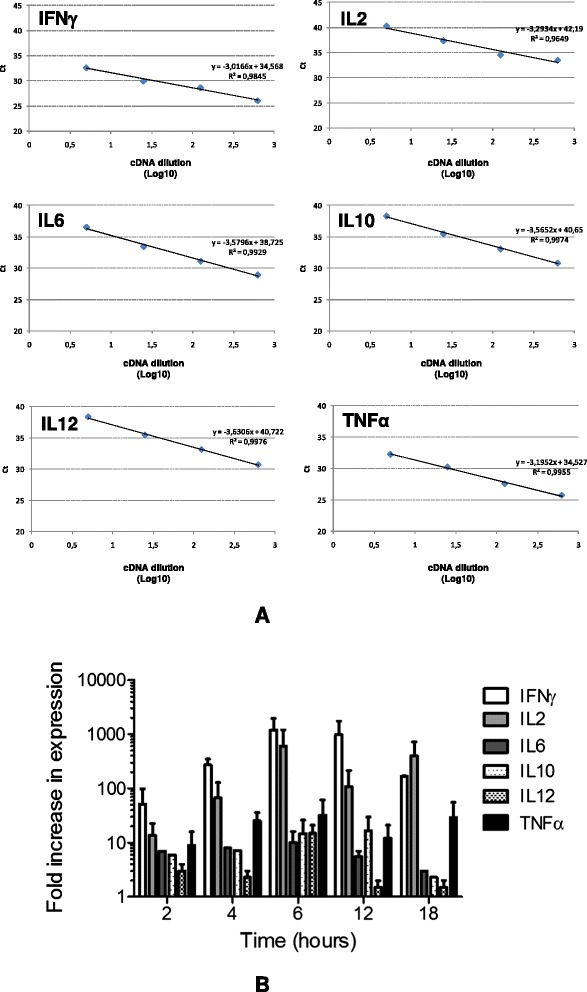
**Efficiency and kinetics of** ***Saimiri sciureus*** **cytokine cDNA amplification. (A)** Efficiency of cytokine (IFNγ, IL2, IL6, IL10, IL12, TNFα) cDNA amplification by real-time PCR using pair of primers specifically designed for *Saimiri sciureus* sequences. The Figure shows the average Ct values after cytokine amplification using serial five-fold dilutions of cDNA from *Saimiri* peripheral blood mononuclear leukocytes stimulated with ionomycin-PMA. **(B)** Kinetics of cytokine (IFNγ, IL2, IL6, IL10, IL12, TNFα) cDNA amplification by real-time PCR following in vitro mitogen stimulation. Peripheral blood mononuclear leukocytes from non-infected *Saimiri* were stimulated in vitro with ionomycin-PMA and mRNA amplification was determined at various timepoints. Results are shown as fold increases in cytokine expression in relation to unstimulated leukocytes.

The partial sequences of 13 cytokine genes from *S. sciureus* and *A. infulatus* had been previously described [14]. Based on these sequences, primers for qRT-PCR were designed for IFNγ, TNFα, IL2, IL6, IL10 and IL12, as well as for the housekeeping gene β-actin, used to normalize expression levels. All primers amplified the corresponding cDNA fragment obtained from *Saimiri* PBMC stimulated *in vitro* with mitogens (ionomicyn-PMA) with satisfactory efficiency and specificity (Figure 1A). An assessment of the kinetics of gene expression upon mitogenic stimulation showed increased cytokine mRNA expression in relation to pre-stimulation (time zero) at all time points tested (two to 18 hours), with the highest expression levels observed at six hours for most cytokines tested (Figure 1B).

### Cytokine expression in *Saimiri* splenocytes during and after *Plasmodium falciparum* infection

**Figure 2:**
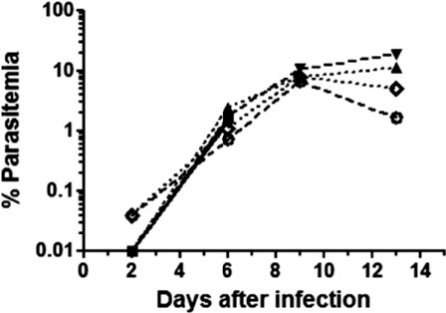
**Course of parasitaemia.** Six *Saimiri sciureus* monkeys were inoculated with 5×10^7^ *P. falciparum* parasitized red blood cells. Two monkeys were splenectomized and treated with chloroquine at d7 (solid lines). The four remaining monkeys were treated with chloroquine at d13, and two of them were splenectomized on day 13 (dashed lines) and two on day 28 (dotted lines).

Six adult *S. sciureus* monkeys were inoculated with 5×10^7^*P. falciparum*-FUP pRBC. All monkeys became parasitaemic, with parasitaemias in the range of 1-2% on d6 (Figure 2). Two animals were splenectomized on d7 and then treated with chloroquine. Parasitaemia continued to grow in the remaining four animals and all were treated on d13. Two of these four monkeys were splenectomized on d13 and the other two on d28 post-infection (15 days after starting chloroquine treatment).

**Figure 3:**
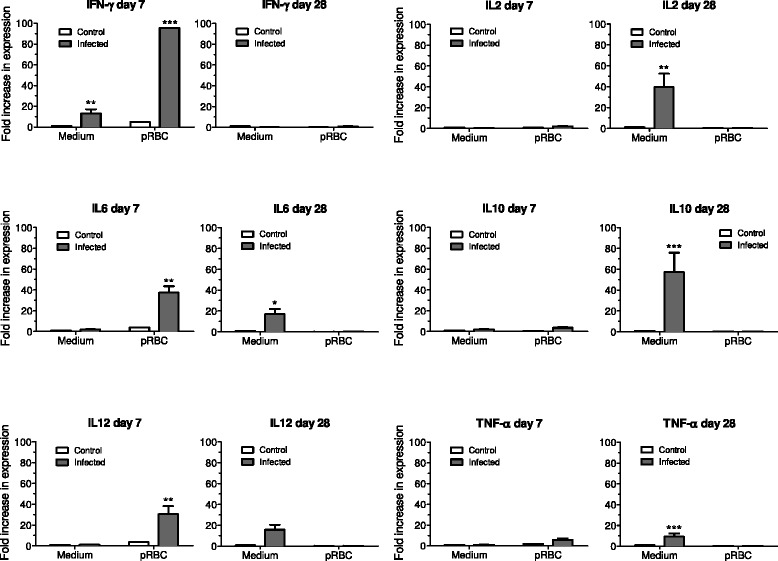
**Cytokine expression by real-time PCR in** ***Saimiri sciureus*** **splenocytes.** Animals were splenectomized at d7 (two monkeys, rising parasitaemia) and d28 (two monkeys, 15 days after start of chloroquine therapy) post-inoculation with 5×10^7^ *P. falciparum* pRBC. Splenocytes were stimulated *in vitro* with *P. falciparum* pRBC or culture medium. Fold increases in cytokine expression were calculated in relation to unstimulated splenocytes from two uninfected control *Saimiri* monkeys. *: p <0.05; **: p <0.01; ***: p <0.001 (two-way ANOVA with Bonferroni post test).

Splenocytes of *P. falciparum*-infected monkeys stimulated with *P. falciparum*-pRBCs showed increased expression levels of IFNγ, IL6 and IL12 on d7 of infection (Figure 3). IFNγ expression, in particular, was increased nearly 100-fold. In addition, IFN-γ was the only cytokine whose expression was increased in non-stimulated splenocytes from *P. falciparum*-infected monkeys. The pattern of cytokine expression was quite different 15 days after the infection was cured (d28 post-inoculation). At this point, stimulation of splenocytes with *P. falciparum* pRBC was unable to induce expression of any of the cytokines studied (Figure 3). However, and interestingly, non-stimulated splenocytes of *P. falciparum*-infected *Saimiri* monkeys showed increased expression of TNFα, IL2, IL6, IL10, and IL12 after six hours of unstimulated *in vitro* culture. Considering the spontaneous increased expression in non-stimulated cells, it can be concluded therefore that adding *P. falciparum* pRBC actually inhibited expression of these cytokines. IFNγ, the only cytokine with increased expression in non-stimulated cells on d7, showed no change in expression on d28.

### Histology

**Figure 4:**
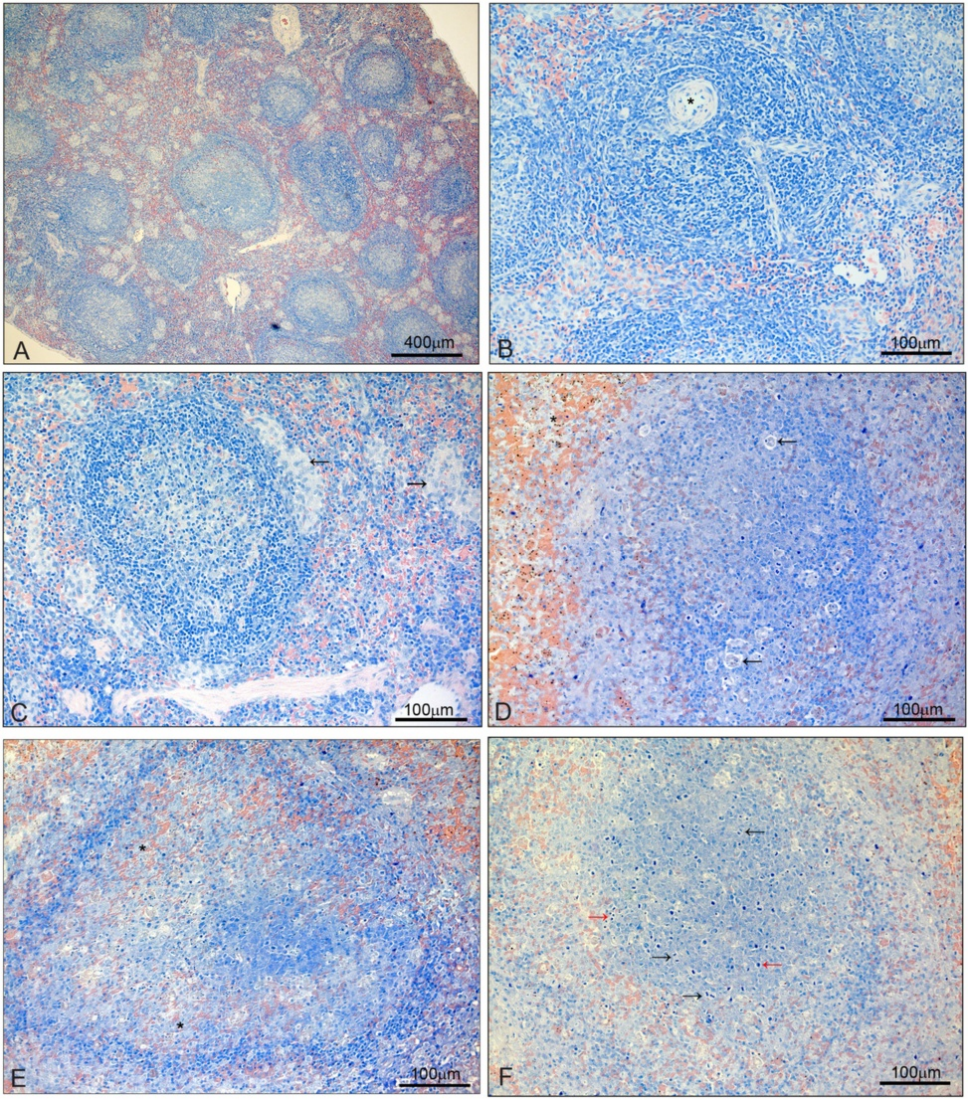
**Changes in spleen histology during** ***Plasmodium falciparum*** **infection. 4A**-**C**: Spleens of non-infected control animals, showing well-defined limits between the red and white pulps **(A)**, quiescent T-cell zones without immunoblasts **(B)**; a central arteriole is denoted by an asterisk) and mostly resting B-cell follicles **(C)**. Clusters of weakly stained cells, suggestive of macrophages, were observed (**A**,** C**, arrows). **D-F**: At d7 of infection, haemozoin was observed throughout an engorged red pulp (**D**, asterisk). Follicles showed numerous phagocytosis centers (**D**, arrows). Limits between the red and white pulps were blurred and follicles presented light-stained nuclei cells surrounded by small lymphocytes **(E)**, and the B-cell follicles showed penetration by RBCs (**E**, asterisks). Large cells with weakly stained nuclei suggestive of centroblasts were widespread (central darker area of the image), and mitosis (black arrows) and apoptosis foci (red arrows) were abundant **(F)**. All sections were stained with Lennert’s Giemsa.

Spleens of non-infected control *Saimiri* monkeys showed quiescent T-cell zones, without immunoblasts (Figure 4A) and mostly resting B-cell follicles. The B follicles commonly presented a central area of weakly stained cells surrounded by small lymphocytes (Figure 4B, C) although a few germinal centres phase I and eventually phase II were observed. Rare mitoses were observed and there were well-defined limits between the red and white pulps (Figure 4A). The red pulp presented mostly cells with weakly stained nuclei, especially near the outer capsule. Interestingly, several clusters of weakly stained cells, suggestive of macrophages, were observed in the spleen of uninfected *Saimiri* monkeys (Figure 4A, C).

On d7 of infection, malaria pigment was observed in pRBCs throughout an engorged red pulp (Figure 4D). Several immunoblasts were found in the T-cell zone. The B-cell follicles showed penetration by RBCs, and limits between the red and white pulps were blurred (Figure 4E). Large cells with weakly stained nuclei suggestive of centroblasts were widespread, and mitosis and apoptosis foci were also abundant (Figure 4F). The characteristic phases of germinal centre development were not observed, and follicles presented typically a wide area of light-stained nuclei cells surrounded by small lymphocytes (Figure 4E).

**Figure 5:**
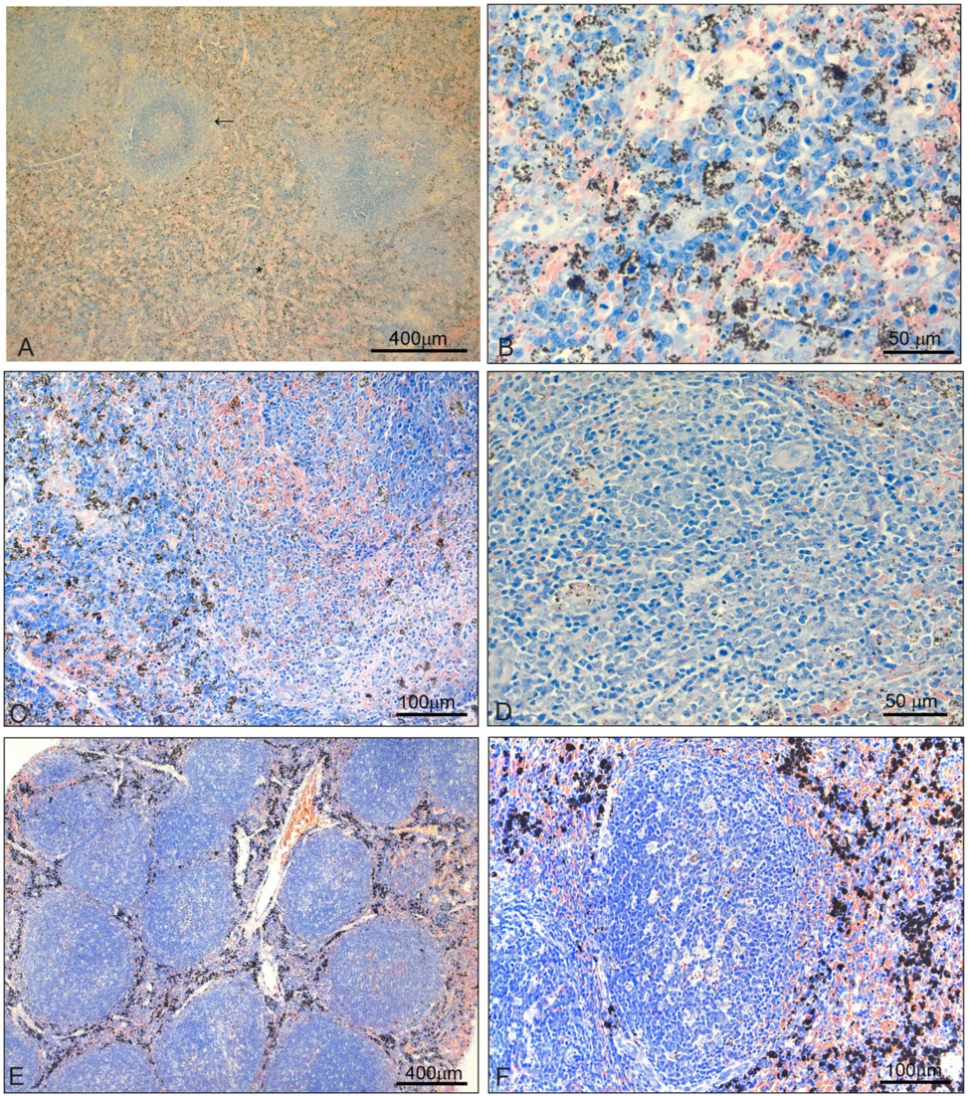
**Changes in spleen histology during** ***Plasmodium falciparum*** **infection. 5A-D:** At d13 of infection (just before chloroquine treatment), the red pulp was further enlarged in relation to controls and d7 of infection and laden with haemozoin (**A**, asterisk). The follicles were also enlarged and limits between the red and white pulp were blurred (**A**, arrow). Phagocytes were heavily laden with pigment and strings of cells suggestive of plasmacytogenesis cords were observed near the T-cell zone **(B)**. Follicles showed penetrating RBCs and limits between red and white pulp were blurred **(C)**. Follicles were interspersed with cells of different morphology, showing phagocytes with malaria pigment, centroblasts and mainly small lymphocytes in the periphery **(D)**.** E**,** F**: At d28 (15 days after start of chloroquine treatment), the red pulp phagocytes were heavily laden with malaria pigment **(E**,** F)**. The distribution of haemozoin-containing phagocytes was better defined in relation to d13, with individual cells containing more compacted, less granulous pigment **(E**,** F)**. The follicles were further enlarged in relation to d13, with large follicles touching each other with little red pulp in between **(E)** and showed mostly activated cells **(F)**. All sections were stained with Lennert’s Giemsa.

On d13 – the day of peak parasitaemia, just before chloroquine treatment – both red and white pulps were enlarged in relation to controls and d7 of infection and phagocytes were heavily laden with pigment (Figure 5A, B). Follicles showed penetrating RBCs and limits between red and white pulp were blurred (Figure 5A, C). Follicles were interspersed with cells of different morphology, showing phagocytes with malaria pigment, centroblasts and mainly small lymphocytes in the periphery (Figure 5D). Strings of cells suggestive of plasmacytogenesis cords [19] were observed near the T-cell zone (Figure 5B).

On d28 (15 days after chloroquine treatment) the red pulp phagocytes were heavily laden with malaria pigment (Figure 5E, F). The distribution of haemozoin-containing phagocytes was better defined in relation to d13, with cells distributed in the red pulp surrounding the follicles, and with individual cells containing more compacted, less granulous pigment (Figure 5E, F). This clear-cut distribution of haemozoin-containing phagocytes in the red pulp without penetrating the follicles indicated a better structured spleen, likely recovering from the architecture disarray peaking on d13. Despite animals had been treated for 15 days and clear of parasites for over ten days, the follicles were further enlarged in relation to d13, with large follicles touching each other with little red pulp in between (Figure 5E) and showed mostly activated cells (Figure 5F).

## Discussion

In this study, primers for quantitative real time PCR for *Saimiri* IFNγ, TNFα, IL2, IL6, IL10, and IL12 were validated and used in a study of splenic responses in *S. sciureus* during blood infection by *P. falciparum*. All primers were shown to amplify their respective target sequences with efficiency and specificity. Although some studies took advantage of human sequences to evaluate cytokine expression in *Saimiri* and *Aotus* [20,21], the availability of primers specifically designed for these species brings more confidence for reliable amplification of the target sequences. Importantly, because each of the described primer sets was designed using sequences common to *Saimiri* and *Aotus*, they can be used to amplify the target sequences from material derived from both species.

Most malaria vaccine studies in these non-human primates use splenectomized animals to ensure consistent and reproducible parasitaemias [10-13,22-27]. Indeed, *P. falciparum* infections in intact, non-splenectomized *Saimiri* monkeys can be unsuccessful or produce short-term, low-grade, variable parasitaemias. However, the present study, as well as previous reports, show that a consistent and substantial parasitaemia can be achieved in non-splenectomized animals using strains better adapted through repeated passages in intact animals. Contamin and colleagues [28] used a *P. falciparum* clone derived from the FUP strain to induce blood stage infection in *S. sciureus* with a relatively low inoculum of 1x10^6^ pRBC. They reported that the course of infection was reproducible, with similar lengths of pre-patent and patent periods and all animals were able to control and cure their parasitaemia without the need for treatment. The peak parasitaemia, however, varied between animals, ranging from 2 to 10%. Horii and colleagues immunized *S. sciureus* with a recombinant protein derived from the *P. falciparum* serine repeat antigen 5 and challenged them with a high inoculum of 1×10^9^ pRBC of the *P. falciparum* Indochina-1/CDC strain [29]. Non-immunized control animals developed a parasitaemia over 10% with a short pre-patent period and were also able to self-cure without the need for treatment. In the present study, a consistent parasitaemia was induced in non-splenectomized animals using an inoculum of 5×10^7^ pRBC of the *P. falciparum* uncloned FUP strain. The spleen is a key organ in immune response against malaria and an important site of parasite killing by macrophages [30-36]. In addition, splenectomy makes the model more distant of the human malaria characteristics. Therefore, the use of intact animals, besides making the model closer and more relevant for human malaria, provides a unique opportunity to study splenic immune responses during falciparum malaria.

The profile of cytokine expression of splenocytes upon pRBC stimulation *in vitro* showed interesting features. First, during the acute phase of infection, on d7, when parasitaemia is growing but relatively low, IFNγ was the cytokine with the highest expression levels, followed by IL6 and IL12. On the other hand, IL2, IL10 and TNFα showed no changes in expression in relation to unstimulated cells from uninfected control monkeys. This pattern indicates that blood stage *P. falciparum* induces a potent Th1-skewed response in *S. sciureus*. In humans, cytokines such as IFNγ and IL12 enables the host to effectively manage the exponential growth of *Plasmodium* until an effective adaptive response is established [37]. In addition, a pro-inflammatory type of response is associated with more rapid control of parasite growth but also with the development of clinical symptoms [38]. In mice, blood stage plasmodial infections also usually induce an early Th1-type response. A switch later to a predominant Th2 response is associated with acquisition of immunity, while the persistence of the Th1 response may generate immunopathology [39]. The evaluation of Th2 responses in this study was limited by the availability of primers for IL10 only. It is important to develop primers for IL4, IL13 and TGFβ, for example, and also other pro-inflammatory and regulatory cytokines, such as IL17 and IL18, for better and more complete assessment of the cytokine expression profile. It is known that the expression of anti-inflammatory cytokines such as IL4 and IL10 is critical in the control of anaemia and severe malaria and the expression of these cytokines is associated with biochemical patterns of iron deficiency in infected children [40]. *Saimiri* and *Aotus* are vulnerable to one of the most serious complications of malaria, severe anaemia [10-13] and, therefore, these models could be explored to study the cytokine response in the context of this malaria complication.

The elevated levels of IFNγ, IL6 and IL12 on d7 of infection were mirrored histologically by the presence of haemozoin-containing phagocytes, several immunoblasts (blast T cells) in the peri-arteriolar lymphatic sheath area and B-cell activation (centroblasts) in germinal centres. Technical difficulties associated with splenocyte isolation on d13 precluded the analyses of the cytokine expression profiles at this important time point. At this stage, the spleen showed evidence of strong and disorganized B cell activation and proliferation, and phagocytes were heavily laden with haemozoin.

Splenocytes of *Saimiri* monkeys on d28 (15 days after the start of anti-malarial treatment) showed a very different response pattern in relation to early, acute infection (d7). Cells incubated for six hours without any antigenic stimulation showed increased baseline expression of IL2, IL6, IL10, IL12, and TNFα, but not IFNγ. Therefore, 15 days after start of treatment, splenocytes remained activated. In addition, a relevant change in the profile of cytokine expression was observed in relation to the acute phase of infection, with IL10 being switched on and IFNγ off. Strikingly, contact with the parasite *in vitro*, instead of stimulating a further burst in cytokine expression, actually shut down the splenocyte response for all cytokines. One possible explanation for this phenomenon is that cells strongly stimulated *in vivo* by infection are activated, proliferate and differentiate to a state at which they were no longer able to be restimulated [41]. Instead, the shut down in the response suggests that active mechanisms of immunosuppression, such as antigen-induced anergy [42] or induction of apoptosis [43,44], are actually taking place.

It has indeed been shown that malaria parasites have mitogenic properties and injection of polyclonal B cell activators prior to immunization of normal mice may suppress the response to antigens [45-47]. This phenomenon may be one of the factors involved in the genesis of immunosuppression associated with human and experimental malarias [44,45]. The histological findings support this line of interpretation, as the white pulp was much enlarged in relation to uninfected controls, with extensive predominance of activated cells over small lymphocytes. Importantly, macrophages heavily laden with haemozoin persisted even 15 days after treatment. Haemozoin has been shown to suppress immune responses *in vitro* [48-50], therefore the inability of the spleen to eliminate it is probably responsible for the persistent cellular activation long after treatment and a key factor involved in the malaria-driven malfunction of the immune system.

Persistent activation was also evident in the B cell compartment. Follicle disarray with disturbance of germinal centre architecture responses in *Saimiri* was similar to the findings reported in human [34] and murine (*Plasmodium berghei*, *Plasmodium chabaudi*) malarias [19,51] and therefore it appears to be an universal feature of malaria infections. Follicles remained enlarged and composed mainly by activated B cells 15 days after the start of chloroquine treatment, which is consistent with a status of spontaneous polyclonal B cell activation previously demonstrated both in rodent and human malaria [52-54].

Altogether, these observations may have fundamental importance for the understanding of the mechanisms of immunity to malaria and for vaccine development. Indeed, a potential vaccine may fail in the field due to persistently compromised immune systems of malaria-exposed individuals rather than to any intrinsic flaws in its immunogenic and protective features.

## Conclusion

The present study shows that quantitative real-time PCR using primers designed for *Saimiri* and *Aotus* cytokines can be valuable tools in immunological research using these primate models, particularly in malaria vaccine and pathogenesis studies. In addition, the patterns of cytokine expression during *P. falciparum* blood stage growth and after anti-malarial treatment in *Saimiri* indicates that blood stage infection induces an early Th1-skewed immune response, that splenocytes of infection-primed animals remain activated after cure probably due to haemozoin persistence, and that antigen stimulation impairs rather than boosts cytokine response in the spleen.

## References

[1] Baird JK, Sismadi P, Masbar S, Ramzan A, Purnomo BW, Sekartuti A (1996). A focus of endemic malaria in Central Java. Am J Trop Med Hyg.

[2] Baird JK, Agyei SO, Utz GC, Koram K, Barcus MJ, Jones TR (2002). Seasonal malaria attack rates in infants and young children in northen Ghana. Am J Trop Med Hyg.

[3] Perlmann P, Troye-Blomberg M (2002). Malaria immunology. Folia Parasitol.

[4] Langhorne J, Ndungu FM, Sponaas AM, Marsh K (2008). Immunity to malaria: more questions than answers. Nat Immunol.

[5] Williamson WA, Greenwood BM (1978). Impairment of the immune response to vaccination after acute malaria. Lancet.

[6] Artavanis-Tsakonas K, Tongren JE, Riley EM (2003). The war between the malaria parasite and the immune system: immunity, immunoregulation and immunopathology. Clin Exp Immunol.

[7] van der Heyde HC, Nolan J, Combes V, Gramaglia I, Grau GE (2006). A unified hypothesis for the genesis of cerebral malaria: sequestration, inflammation and hemostasis leading to microcirculatory dysfunction. Trends Parasitol.

[8] Carvalho LJ, Daniel-Ribeiro CT, Goto H (2002). Malaria vaccine: candidate antigens, mechanisms, constraints and prospects. Scand J Immunol.

[9] World Health Organization (1988). Role of non-human primates in malaria vaccine development: memorandum from WHO meeting. Bull World Health Organ.

[10] Carvalho LJM, Oliveira SG, Alves FA, Brígido MCO, Muniz JAPC, Daniel-Ribeiro CT (2000). *Aotus infulatus* monkey is susceptible to *Plasmodium falciparum* and may constitute an alternative experimental model for malaria. Mem Inst Oswaldo Cruz.

[11] Carvalho LJM, Alves FA, Oliveira SG, Valle RR, Fernandes AAM, Muniz JAPC (2003). Severe anemia affects both splenectomized and non-splenectomized *Plasmodium falciparum*-infected *Aotus infulatus* monkeys. Mem Inst Oswaldo Cruz.

[12] Carvalho LJM, Oliveira SG, Theisen M, Alves FA, Andrade MCR, Zanini GM (2004). Immunization of *Saimiri sciureus* monkeys with *Plasmodium falciparum* merozoite surface protein-3 and glutamate rich-protein suggest that protection is related to antibody levels. Scand J Immunol.

[13] Carvalho LJM, Alves FA, Bianco C, Oliveira SG, Zanini GM, Soe S (2005). Immunization of *Saimiri sciureus* monkeys with a recombinant hybrid protein derived from *Plasmodium falciparum* antigen glutamate rich-protein and merozoite surface protein-3 can induce partial protection with Freud and Montanide ISA720 adjuvants. Clin Diagn Lab Immunol.

[14] Alves FA, Souza MT, Gonçalves EC, Schneider MP, Marinho AM, Muniz JA (2010). DNA sequencing of 13 cytokine gene fragments of *Aotus infulatus* and *Saimiri sciureus*, two non-human primate models for malaria. Cytokine.

[15] Trager W, Jensen JB (1976). Human malaria parasites in continuous culture. Science.

[16] Carson FL, Martin JH, Lynn JA (1973). Formalin fixation for electron microscopy: a re-evaluation. Am J Clin Pathol.

[17] Lennert K. Malignant Lymphomas Other than Hodgkin’s Disease. Histology, Cytology, Ultrastructure, Immunology. Handbuch der Speziellen Pathologischen Anatomie und Histologie. 1978;1-71.

[18] Ayres-Silva JP, Manso PP, Madeira MR, Pelajo-Machado M, Lenzi HL (2011). Sequential morphological characteristics of murine fetal liver hematopoietic microenvironment in Swiss Webster mice. Cell Tissue Res.

[19] Carvalho LJM, Ferreira-da-Cruz MF, Daniel-Ribeiro CT, Pelajo-Machado M, Lenzi HL (2007). Germinal center architecture disturbance during *Plasmodium berghei* ANKA infection in CBA mice. Malar J.

[20] Diaz D, Daubenberger CA, Zalac T, Rodriguez R, Patarroyo ME (2002). Sequence and expression of MHC-DPB1 molecules of the New World monkey *Aotus nancymaae*, a primate model for *Plasmodium falciparum*. Immunogenetics.

[21] Kazanji M, Heraud JM, Merien F, Pique C, de Thé G, Gessain A (2006). Chimeric peptide vaccine composed of B- and T-cell epitopes of human T-cell leukemia virus type 1 induces humoral and cellular immune responses and reduces the proviral load in immunized squirrel monkeys (*Saimiri sciureus*). J Gen Virol.

[22] Gysin J, Hommel M, Silva LP (1980). Experimental infection of the squirrel monkey (*Saimiri sciureus*) with *Plasmodium falciparum*. J Parasitol.

[23] Perrin LH, Merkli B, Loche M, Chizzolini C, Smart J, Richle R (1984). Antimalarial immunity in *Saimiri* monkeys. Immunization with surface components of asexual blood stages. J Exp Med.

[24] Fandeur T, Le Scanf C, Bonnemains B, Slomianny C, Mercereau-Puijalon O (1995). Immune pressure selects for *Plasmodium falciparum* parasites presenting distinct red blood cell surface antigens and inducing strain-specific protection in *Saimiri sciureus* monkeys. J Exp Med.

[25] Perraut R, Mercereau-Puijalon O, Mattei D, Bourreau E, Bonnefoy S, Bonnemains B (1997). Immunogenicity and efficacy trials with *Plasmodium falciparum* recombinant antigens identified as targets of opsonizing antibodies in the naive squirrel monkey *Saimiri sciureus*. Am J Trop Med Hyg.

[26] Kumar S, Collins W, Egan A, Yadava A, Garraud O, Blackman MJ (2000). Immunogenicity and efficacy in *Aotus* monkey of four recombinant *Plasmodium falciparum* vaccines in multiple adjuvant formulations based on the 19-kilodalton C-terminal of merozoite surface protein 1. Infect Immun.

[27] Collins WE, Sullivan JS, Williams A, Nace D, Williams T, Galland GG (2006). *Aotus nancymaae* as a potential model for the testing of anti-sporozoite and liver stage vaccines against *Plasmodium falciparum*. Am J Trop Med Hyg.

[28] Contamin H, Behr C, Mercereau-Puijalon O, Michel J (2000). *Plasmodium falciparum* in the squirrel monkey (*Saimiri sciureus*): infection of non-splenectomised animals as a model for exploring clinical manifestations of malaria. Microbes Infect.

[29] Horii T, Shirai H, Jie L, Ishii KJ, Palacpac NQ, Tougan T (2010). Evidences of protection against blood-stage infection of *Plasmodium falciparum* by the novel protein vaccine SE36. Parasitol Int.

[30] Barnwell JW, Howard RJ, Miller LH (1983). Influence of the spleen on the expression of surface antigens on parasitized erythrocytes. Ciba Found Symp.

[31] David PH, Hommel M, Miller LH, Udeinya IJ, Oligino LD (1983). Parasite sequestration in *Plasmodium falciparum* malaria: spleen and antibody modulation of cytoadherence of infected erythrocytes. Proc Natl Acad Sci U S A.

[32] Austin JM, Kupiec-Weglinski JW, Hankins DF, Morris PJ (1988). Migration patterns of dendritic cells in the mouse. Homing to T cell-dependent areas of spleen, and binding within marginal zone. J Exp Med.

[33] Krucken J, Mehnert LI, Dkhil MA, El-Khadragy M, Benten WP, Mossmann H (2005). Massive destruction of malaria-parasitized red blood cells despite spleen closure. Infect Immun.

[34] Urban BC, Hien TT, Day NP, Phu NH, Roberts R, Pongponratn E (2005). Fatal *Plasmodium falciparum* malaria causes specific patterns of splenic architectural disorganization. Infect Immun.

[35] Contini S, Lewis HRN (2006). Spleen abscess as malaria complication. Emerg Infect Dis.

[36] Sponaas AM, Cadman ET, Voisine C, Harrison V, Boonstra A, O’Garra A (2006). Malaria infection changes the ability of splenic dendritic cell populations to stimulate antigen-specific T cells. J Exp Med.

[37] Artavanis-Tsakonas K, Riley EM (2002). Innate immune response to malaria: rapid induction of IFN-gamma from human NK cells by live *Plasmodium falciparum*-infected erythrocytes. J Immunol.

[38] Hensmann M, Kwiatkowski D (2001). Cellular basis of early cytokine response to *Plasmodium falciparum*. Infect Immun.

[39] Perlmann P, Perlmann H, Berzins K, Troye-Blomberg M (1998). Selected problems of malaria blood stage immunity. Tokai J Exp Clin Med.

[40] Nyakeriga AM, Williams TN, Marsh K, Wambua S, Perlmann H, Perlmann P (2005). Cytokine mRNA expression and iron status in children living in a malaria endemic area. Scand J Immunol.

[41] Spira DT, Golenser J, Gery I (1976). The reactivity of spleen cells from malarious rats to non-specific mitogens. Clin Exp Immunol.

[42] Martini F, Paglia MG, Montesano C, Enders PJ, Gentile M, Pauza CD (2003). V gamma 9 V delta 2 T-cell anergy and complementarity-determining region 3-specific depletion during paroxysm of nonendemic malaria infection. Infect Immun.

[43] Dey S, Guha M, Alam A, Goyal M, Bindu S, Pal C (2009). Malarial infection develops mitochondrial pathology and mitochondrial oxidative stress to promote hepatocyte apoptosis. Free Radic Biol Med.

[44] Wilson NO, Huang MB, Anderson W, Bond V, Powell M, Thompson WE (2008). Soluble factors from *Plasmodium falciparum*-infected erythrocytes induce apoptosis in human brain vascular endothelial and neuroglia cells. Mol Biochem Parasitol.

[45] Wyler DJ, Herrod HG, Weinbaum FI (1979). Response of sensitized and unsensitized human lymphocyte subpopulations to *Plasmodium falciparum* antigens. Infect Immun.

[46] Ballet JJ, Druilhe P, Querleux MA, Schmitt C, Agrapart M (1981). Parasite-derived mitogenic activity for human T cells in *Plasmodium falciparum* continuous cultures. Infect Immun.

[47] Diamantstein T, Keppler W, Blitstein-Willinger E (1976). Suppression of the primary immune response *in vivo* to sheep red blood cells by B-cell mitogens. Immunology.

[48] Millington OR, Di Lorenzo C, Phillips RS, Garside P, Brewer JM (2006). Suppression of adaptive immunity to heterologous antigens during *Plasmodium* infection through hemozoin-induced failure of dendritic cell function. J Biol.

[49] Wykes MN, Good MF (2008). What really happens to dendritic cells during malaria?. Nat Rev Microbiol.

[50] Schwarzer E, Alessio M, Ulliers D, Arese P (1998). Phagocytosis of the malarial pigment, hemozoin, impairs expression of major histocompatibility complex class II antigen, CD54, and CD11c in human monocytes. Infect Immun.

[51] Sanchez-Torres L, Rodriguez-Ropon A, Aguilar-Medina M, Favila-Castillo L (2001). Mouse splenic CD4+ and CD8+ T cells undergo extensive apoptosis during a *Plasmodium chabaudi chabaudi* AS infection. Parasite Immunol.

[52] Rosenberg YJ (1978). Autoimmune and polyclonal B cell responses during murine malaria. Nature.

[53] Banic DM, Viana-Martins FS, De Souza JM, Peixoto TD, Daniel-Ribeiro C (1991). Polyclonal B-lymphocyte stimulation in human malaria and its association with ongoing parasitemia. Am J Trop Med Hyg.

[54] Rolland L, Ballet JJ, Daniel-Ribeiro C (1992). Kinetics of antigen specific and non-specific polyclonal B-cell responses during lethal *Plasmodium yoelii* malaria. Mem Inst Oswaldo Cruz.

